# VRSPi: towards a neuroadaptive VR exposure therapy system for spider phobia

**DOI:** 10.3389/fnhum.2026.1717588

**Published:** 2026-03-04

**Authors:** Selina C. Wriessnegger, Suktipol Kiatthaveephong, Michael Leitner, Kyriaki Kostoglou

**Affiliations:** 1 Institute of Neural Engineering, Graz University of Technology, Graz, Austria; 2 School of Information Science and Technology (IST), Vidyasirimedhi Institute of Science and Technology (VISTEC), Rayong, Thailand

**Keywords:** arachnophobia, EEG, heart rate, neuroadaptive system, virtual reality, VRET

## Abstract

**Introduction:**

Arachnophobia, the fear of spiders, is one of the most common phobias globally that induce anxiety and other mental illnesses if left untreated. Virtual reality (VR)-based exposure therapy (VRET) has recently emerged as a viable solution for treating different phobias. In this work, we present a proof-of-concept study toward the development of a neuroadaptive and individualized VRET system (VRSpi) that integrates virtual reality with real-time neurophysiological monitoring, intended as a future tool for personalized arachnophobia treatment. VRSpi is designed to monitor brain and heart rate (HR) responses in real-time to automatically control the intensity of the fear stimulus in an adaptive manner.

**Methods:**

Twenty-one healthy participants not clinically diagnosed with arachnophobia attended the study, indicating no or moderate fear of spiders. The moderate fear group tested the efficacy of our system towards treating their arachnophobia. Since fear-related emotions are primarily processed in the right frontal hemisphere of the brain, the study used the frontal alpha asymmetry (FAA) index from frontal electroencephalography (EEG) channels, along with HR, to automatically assess the participant’s fear level during VR spider exposure.

**Results:**

We could demonstrate that incorporating FAA index measurements could improve personalization in VRET by allowing the VR environment to automatically adapt to each individual’s fear level, potentially improving treatment effectiveness, though its direct impact on clinical outcomes require further study. Moreover, our findings indicate that escalating spider scene exposure is associated with reduced oscillatory power in faster bands (alpha, beta, and gamma) and increased frontal theta activity, reflecting a neural state characterized by heightened vigilance, emotional reactivity, and regulatory effort. In addition, these effects were accompanied by systematic right-frontal EEG shifts, characteristic HR changes, and reliable discrimination between low and high fear states.

**Discussion:**

Future work should address the clinical integration of neuroadaptive exposure systems. In practice, EEG-based adaptations must be reliable, stable across sessions, and robust to artifacts so therapists can use them as decision-support tools rather than replacements for clinical judgment. Conservative thresholds, transparent logic, and clinician override options may be essential. While this work focuses on feasibility, these practical factors are key to translating neuroadaptive VRET into usable therapies.

## 1 Introduction

One of the most common specific phobias worldwide is the fear of spiders, also known as arachnophobia. According to Schmitt and Müri (2009), its prevalence is approximately 3.5 to 6.1%, with a higher proportion of women affected. People with arachnophobia often experience typical panic attack symptoms when confronted with a spider or spider-related environments, such as a room with webs or a cellar. These symptoms include increased heart rate (HR), sweating, rapid breathing, feelings of disgust, or a flight instinct. It is believed that most individuals develop their arachnophobia during childhood or adolescence.

This phobia can significantly impact daily life, leading to avoidance behaviors such as refraining from outdoor activities or entering cellar rooms. The primary treatment for arachnophobia is standard exposure therapy (Albakri et al., 2022). In this approach, individuals are initially exposed to their fear through pictorial representations of spiders, such as images and videos. Once the patient feels sufficiently comfortable, real interaction with a live spider is introduced, where they try to minimize the distance to the spider and may even attempt to touch it.

### 1.1 Virtual reality exposure therapy

An increasingly popular alternative is virtual reality exposure therapy (VRET). In VRET, the individual confronts their phobia within a computer-generated, three-dimensional virtual environment. This environment creates a feeling of presence, simulating being physically present in the virtual space, by adapting in real-time to the user’s head movements via head-mounted displays (HMDs). The system also employs sound source control and, occasionally, hand trackers to enable interaction with virtual objects.

While VR has gained popularity in the entertainment industry, its use in medicine is rapidly expanding (Safikhani et al., 2021). It is employed as a learning and skill-enhancement tool for medical students and professionals, as well as a therapeutic modality in rehabilitation and mental health treatment, including VRET. Unlike standard exposure therapy, VRET offers several advantages: it avoids putting patients in real, potentially dangerous or highly stressful situations, making the process more tolerable. Additionally, VR tends to evoke a more realistic and immersive experience compared to the 2D images used in traditional therapy, thus inducing a fear response that more closely resembles real-life scenarios.

Specifically, VRET has been widely validated as an effective treatment for *acrophobia* (fear of heights), facilitating graded, controlled exposure to height-related stimuli that reduces anxiety and avoidance behaviors comparable to traditional *in vivo* exposure paradigms. Studies using immersive VR scenarios (e.g., virtual canyon walkways or high-rise environments) have shown significant reductions in subjective fear and physiological arousal after repeated sessions (Francová et al., 2025), with VR sessions often achieving similar therapeutic gains in less time than real-world exposures (Coelho et al., 2009; Balan et al., 2019; Azimisefat et al., 2022).

Beyond this traditional VRET, neuroadaptive integrations using electroencephalography (EEG) are an emerging frontier in augmenting exposure therapy. Recent work on EEG-based adaptive VR systems demonstrates the feasibility of real-time acquisition and processing of brain signals within XR environments to classify fear intensity and adjust exposure parameters accordingly, potentially enhancing personalization and treatment outcomes by linking neural markers directly to therapy control logic (Apicella et al., 2024). This approach aims to tailor stimulus intensity based on the user’s neural and autonomic state, allowing exposure to progress at a pace aligned with moment-to-moment fear responses, thereby optimizing desensitization while minimizing excessive distress.

Building on this, adaptive/closed-loop VR frameworks adjust exposure parameters (e.g., height, motion, proximity to edges) using online measures such as heart rate, skin conductance, and EEG-derived indices, aiming to keep arousal within a therapeutic “window” that maximizes learning while minimizing dropout (Maples-Keller et al., 2017). For anxiety detection, state-of-the-art systems use multimodal wearables plus machine learning to infer anxiety episodes from physiological signatures; recent systematic reviews summarize signals, feature sets, and model performance, while also emphasizing real-world validity limits and context dependence (Elgendi et al., 2026). Finally, experimental enhancement strategies pair VRET with non-invasive brain stimulation (e.g., tDCS) in a neuromodulation-augmented loop to potentially accelerate extinction learning (Hui et al., 2024).

Beyond their therapeutic potential, VR-based interventions offer practical advantages for clinical deployment. Therapists can maintain better control over the stimuli presented, allowing for more individualized treatment. It also lowers therapy costs by eliminating the need for real spiders and enables telemedicine approaches, where patients can undergo therapy at home with remote guidance and therapist supervision. However, a minor disadvantage of VRET is that some individuals may experience motion sickness during sessions—characterized by eye fatigue, disorientation and nausea (Chang et al., 2020). This VR sickness results from a sensory conflict between perceived orientation and actual movement. Despite this, multiple studies (Carl et al., 2019; Morina et al., 2015; Fodor et al., 2018) suggest that VRET is at least as effective as traditional exposure therapy. Confronting the patients with the feared object under safe controlled conditions is more practical and also reduces traumatic experiences and logistical efforts.

### 1.2 EEG correlates of fear

Several studies (Wang and Wang, 2021) have demonstrated that EEG signals are strongly influenced by emotions, making them useful for emotional recognition. Compared to other physiological measurements, EEG generally offers higher classification accuracy and faster response times to emotional changes. Emotions such as happiness and sadness are primarily processed in the frontal cortex of the brain (Houssein et al., 2022). Further research (Mouri et al., 2023) has also revealed a lateralization effect in processing positive emotions (like happiness) and negative emotions such as sadness.

One of the pioneering researchers in this area, Richard J. Davidson, introduced the concept of hemispheric lateralization during emotional processing and the frontal alpha asymmetry (FAA) index. In his study (Davidson and Tomarken, 1989), participants viewed short film clips eliciting contrasting emotions, such as happiness and disgust. The findings indicated that positive emotions activate the left frontal hemisphere, while negative emotions activate the right frontal hemisphere.

The FAA index quantifies frontal lateralization during emotional stimuli by comparing alpha wave activity (8–13 Hz) in the frontal cortex—using electrodes such as F3 and F4. It is calculated by subtracting the natural logarithm of the alpha power in the left hemisphere from that in the right. A positive FAA index indicates greater left hemisphere activation (lower left alpha power), since decreased alpha power correlates with increased brain activation. Conversely, a negative FAA index suggests higher right hemisphere activation.

Within the motivational direction framework, greater right-frontal cortical activation has been associated with withdrawal-related emotions such as fear and anxiety, whereas greater left-frontal activation is linked to approach-related affective states (Davidson and Tomarken, 1989; Davidson et al., 2000; Coan and Allen, 2004). Because alpha power is inversely related to cortical activation, negative FAA values are therefore expected during fear-inducing or anxiety-provoking situations. This theoretical framework provides a strong rationale for using FAA as a neurophysiological marker of fear during exposure-based paradigms.

In the context of fear, a negative FAA index is expected due to increased right hemisphere activation. Early FAA studies involved exposing participants to emotionally charged images, particularly from the International Affective Picture System (IAPS), or short films designed to elicit specific emotions (Wiedemann et al., 1999; Zhao et al., 2018). Subsequent research explored whether the FAA index could reflect feelings of fear and differ between anxious and non-anxious individuals. For example, Davidson et al. (2000) examined the FAA of individuals with social phobia preparing to give a public speech. They found significantly more negative FAA values in the socially phobic group compared to controls, indicating increased right hemisphere activation associated with anxiety. This confirmed that the FAA index could distinguish anxious individuals in stressful situations.

Only a few studies have integrated EEG measurements into VRET to better assess fear levels. EEG provides a physiological measure linked to fear, enabling more accurate fear detection than subjective assessments, which can be unreliable due to patient self-report bias. Other physiological measures used include HR, HRvariability (HRV), galvanic skin response (GSR), pupil diameter, electromyography (EMG), fear-potentiated startle, blood pressure, and skin temperature.

Basbasse et al. (2022) used a mobile EEG to measure FAA index values during a virtual walk across a skyscraper plank, with participants experiencing different acrophobia levels. They also collected subjective fear ratings and performed a traditional IAPS task. Results showed that only during the VR task FAA index values showed significant changes between fear and neutral conditions, correlating positively with subjective fear. Taking this a step further, Apicella et al. (2024) investigated EEG-based classification of fear responses to increasing elevations in a VR environment. Their findings indicated that the frontal brain regions’ high-beta and gamma activity provided the best discrimination of fear levels. They also identified three levels of fear of heights in acrophobic subjects using EEG and electrocardiographic (ECG) signals. Participants were exposed to increasing virtual heights in a canyon scenario, and their fear was measured using the Acrophobia Questionnaire, Subjective Unit of Distress, and State–Trait Anxiety Inventory. As a result four classifiers i.e., Support Vector Machines (SVM), Deep Neural Networks (DNN), Random Forests (RF), and the k-Nearest Neighbor algorithm (k-NN), were evaluated and the tests showed highest accuracy with a DNN. Together, these studies demonstrate the feasibility of using neurophysiological signals to adapt exposure intensity in immersive environments.

### 1.3 EEG correlates related to fear of spiders

Similar findings are reported in studies focusing on the neural correlates of “fear of spiders”. They highlighted specific neural and electrophysiological responses associated with spider phobia. For example, patients with spider phobia show enhanced late positive potential (LPP) amplitudes when viewing spider images, indicating increased emotional engagement and attentional allocation (Soares et al., 2017). Others reported increased theta and alpha activity exposure to spider stimuli, possibly reflecting emotional processing and fear regulation efforts (Bornas et al., 2009, Ebato et al., 2025). Grassini et al. (2020) investigated hemispheric differences in frontal LPP (f-LPP asymmetry) andFAA in response to images of snakes, spiders, butterflies, and birds. Fearful stimuli like snakes and spiders evoked a large late f-LPP (but not FAA) over the right-frontal hemisphere compared to non-fearful stimuli like birds and butterflies. Also Zhang et al. (2021) investigated the effects of a VRET on spider fear with EEG. The results showed that both spider and neutral images elicited significant LPP and early posterior negativity (EPN) magnitudes. Following therapy, all participants exhibited a reduction in their fear of spider symptoms. They suggested that in-vivo and VRET have comparable effects (Zhang et al., 2021). Balan et al. (2019) employed EEG, GSR, and HR data with DNNs to predict the next level in VR height exposure for acrophobic participants, aiming for real-time adaptive therapy. The first DNN was trained to classify the participant’s current fear state, either on a binary scale (‘relaxation’ vs. ‘fear’) or on a four-level scale (‘relaxation,’ ‘low fear,’ ‘moderate fear,’ ‘high fear’). The output of this network, together with the participant’s physiological data, was then used as input to a second DNN, which determined the next VR height exposure level. After training the DNNs, the participants engaged in the VR task, during which the DNNs automatically adjusted the game level based on physiological responses. To validate the first DNN’s performance, participants also provided self-assessed fear ratings after each completed level. All in all, they achieved an accuracy rate of 73% for the 2-choice fear level scale and a rate of 42% for the 4-choice fear level scale. In an EEG study by Wiens et al. (2022) persons with spider phobia received either in-vivo exposure therapy or VRET. EEG was recorded while participants viewed spider images as well as positive, negative, and neutral pictures. Results indicated that, prior to treatment, patients rated spiders as more negative than control subjects and showed increased EPN and LPP responses to spider images. In the post-treatment, the negative emotional ratings towards spiders were significantly reduced. Kisker et al. (2021) created a system utilizing a fear-inducing VR-cave environment to explore physiological biomarkers of fear, specifically HR and EEG within a highly immersive setting. While EEG signals effectively capture the neural manifestations of fear, HR serves as a peripheral physiological indicator of fear. By combining EEG and HR signals, the system can provide a more reliable representation of fear through multi-level monitoring.

### 1.4 Fear level classification

Detecting different fear levels via biosignals requires a suitable classifier and feature extraction strategy. Arsalan and Majid (2021) demonstrated a strategy using power spectral density (PSD) based features with random forests (RF) and achieved accuracies up to 87.69% in two classes and 83.07% in three classes. Aldayel and Al-Nafjan (2024) extracted PSD and discrete wavelet transform features in combination with RFs, furthermore exploring AdaBoost, SVM, linear discriminant analysis and k-NN. They as well achieved high accuracies, in particular 87.5%, with RFs outperforming all other classifiers regarding precision and recall. This provides a solid foundation for the classification of different fear stages using established classifiers and well-known features, also suggesting RF and SVM as recommended strategies in combination with PSD based features.

### 1.5 Motivation

To the best of our knowledge, no comprehensive, individualized, neurophysiology-integrated VRET system is currently available for real-time arachnophobia treatment. To address this gap, the primary aim of the present work was to design an intelligent, neuroadaptive, and individualized VRET system, termed VRSpi. This system is designed to deliver adaptive and progressive exposure therapy for the treatment of arachnophobia. The key components include: (1) the development of serious game-based VR environments featuring spider stimuli, and (2) an online closed-loop strategy powered by neurophysiological indices (EEG and HR) for adaptive control of fear-related stimulus parameters, such as spider size and number. A pilot single-session trial with 13 healthy individuals reporting moderate fear of spiders has already been published elsewhere (Weber et al., 2024) to evaluate the efficacy of the system. In the present paper, we focus on the neurophysiological results and their correlations with subjective fear states recorded during real-time VRET for arachnophobia using our VRSpi. By combining self-report measures, HR indices, and FAA, we aimed to characterize how fear responses evolve with increasing exposure intensity.

## 2 Materials and methods

### 2.1 Participants

Twenty-one healthy right-handed volunteers participated in this study, consisting of 9 females and 12 males, aged between 23 and 33 years (mean: 27.4; SD:3.2). None of the participants had a clinical diagnosis of arachnophobia or other anxiety disorders. The classification of participants according to their level of spider fear, based on the Fear of Spiders Questionnaire (FSQ), is described in detail in Section 2.2.3. All participants were fully informed about the experimental procedure and given ample time to ask questions. All provided voluntary written consent. During the experiment, they were instructed to sit in a relaxed position and minimize eye and body movements to prevent unwanted artifacts. The study received approval from the ethics committee of Graz University of Technology (GZ EK32/2024).

### 2.2 Devices

#### 2.2.1 VR headset and environment

**Figure 1:**
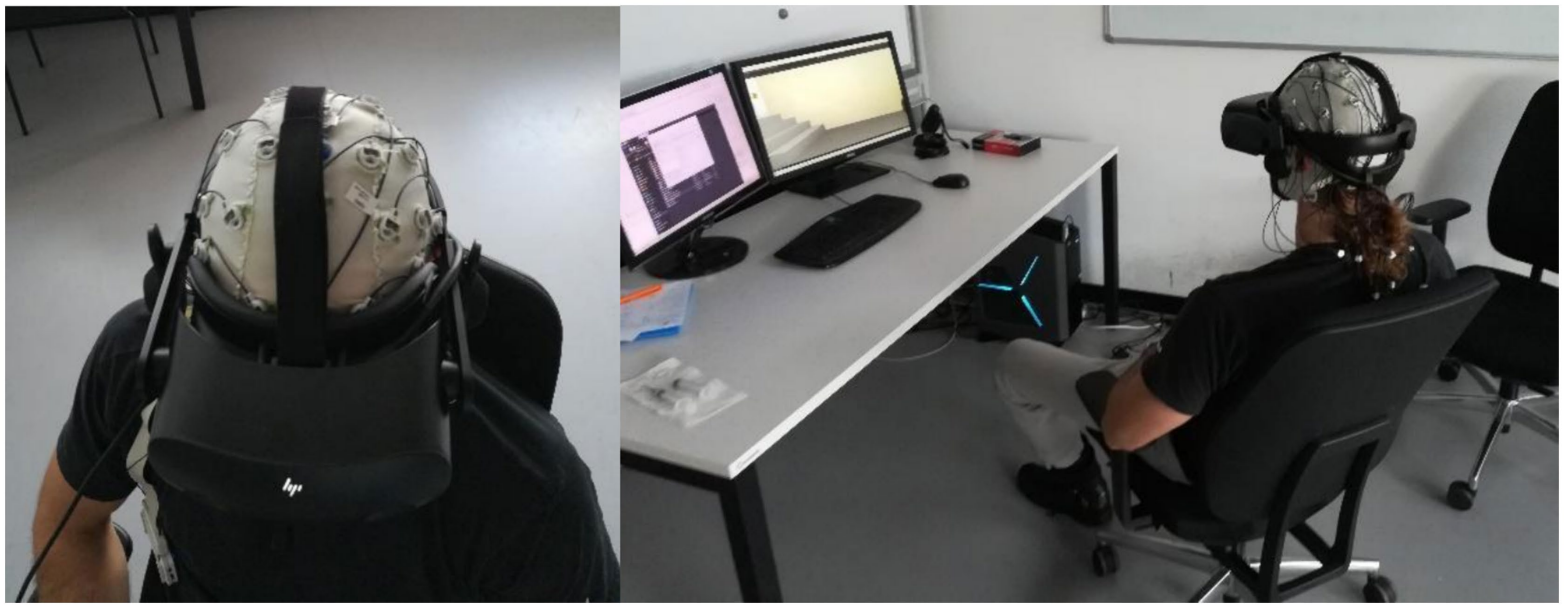
Experimental setup showing a participant wearing a 32-channel EEG cap and the HP Reverb G2 Omnicept Edition head-mounted display during the virtual reality exposure task.

The VR HMD used was the Hewlett Packard Reverb G2 Omnicept Edition (HP Development Company, California, United States), which includes several sensors, such as eye-tracking and HR sensor (Figure 1). Additionally, it features two high-quality speakers positioned on both sides of the VR headset.

**Figure 2:**
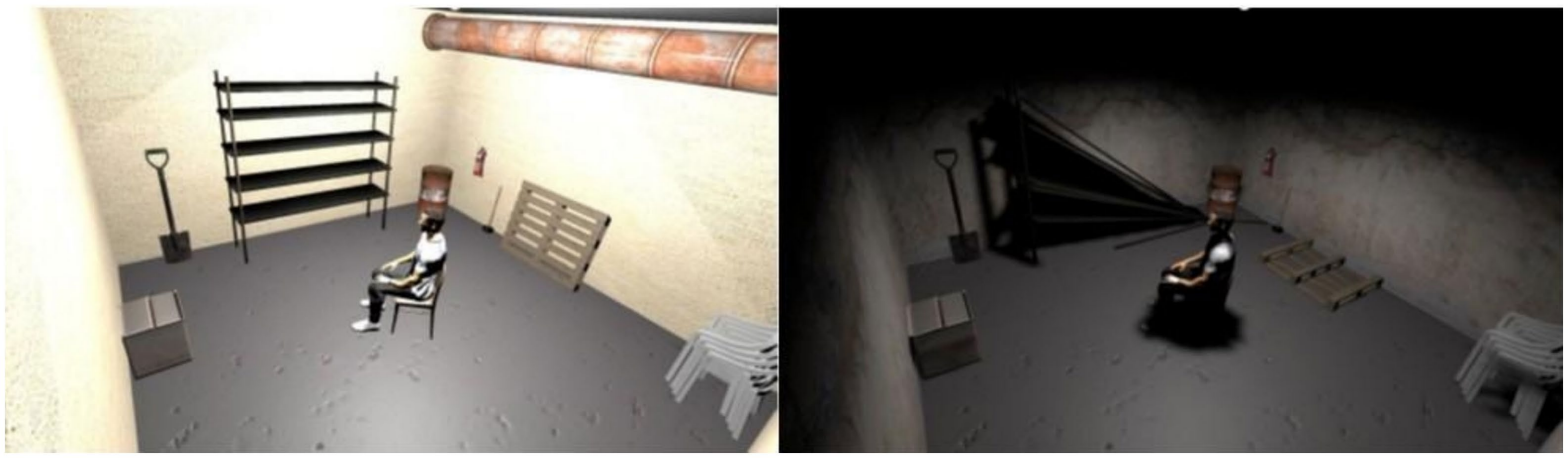
VR scenes used in the experimental paradigm. The neutral VR scene (left) served as a baseline condition, while the spider VR scene (right) represented the fear-inducing environment. The spider scene included visual elements such as spiders and webs, as well as altered lighting and textures, to increase environmental threat and emotional salience.

The VR environment was developed using the Unity game engine (Version 2020.3.25f1). It consists of a virtual basement constructed as a square room with wall textures sourced from the Unity Asset Store pack “18 High Resolution Wall Textures” (A Dog’s Life Software, 2016) (Figure 2). Since two different scenes were required—one neutral and one fear-inducing with spiders—distinct wall textures were used for each: Neutral Scene: Bright, facade-like wall textures to create a neutral atmosphere. Fear Scene: A crumbling, dirty wall texture to evoke an unsettling, cold impression of the room. The floor and ceiling in this scene also featured concrete textures. To enhance realism, a self-designed concrete staircase leading to a door was incorporated into the scene, symbolizing an underground basement. Additionally, various room objects were added to the basement for example, the shelf had both a normal and a broken version, and the barrel was available in a rusty appearance. This allowed for scene-specific variations, providing either standard or fear-inducing appearances for each object.

The spider scene also included multiple spider webs, created using Blender (Blender Foundation, Amsterdam, Netherlands) and strategically placed at the edges of the room to heighten the participant’s sense of a spider-infested environment.

To enhance the participants’ immersive VR experience, a first-person character was added to the scene. Research by Denisova and Cairns (2015) has demonstrated that experiencing the scene from a first-person perspective makes it easier for users to accept the virtual environment as real. The first-person character was positioned sitting on a chair, with hands resting on the thighs to mimic the participant’s natural posture. Based on the participant’s gender, the corresponding male or female character was selected in the Unity editor, while the other was hidden from view. The main camera was placed at the character’s head position, and the head scale was set to a very small value to make the character’s head invisible to the user, avoiding visual distractions. To provide appropriate lighting, two types of lights were used: a point light source for the neutral scene and a spotlight for the fear scene providing also a flickering effect by randomly changing the light’s intensity over time. Additionally, to simulate a realistic light bulb, a sphere object was added to the scene with a special material that had active emission, making the bulb appear as if it was emitting light.

#### 2.2.2 EEG/HR recordings and preprocessing

**Figure 3:**
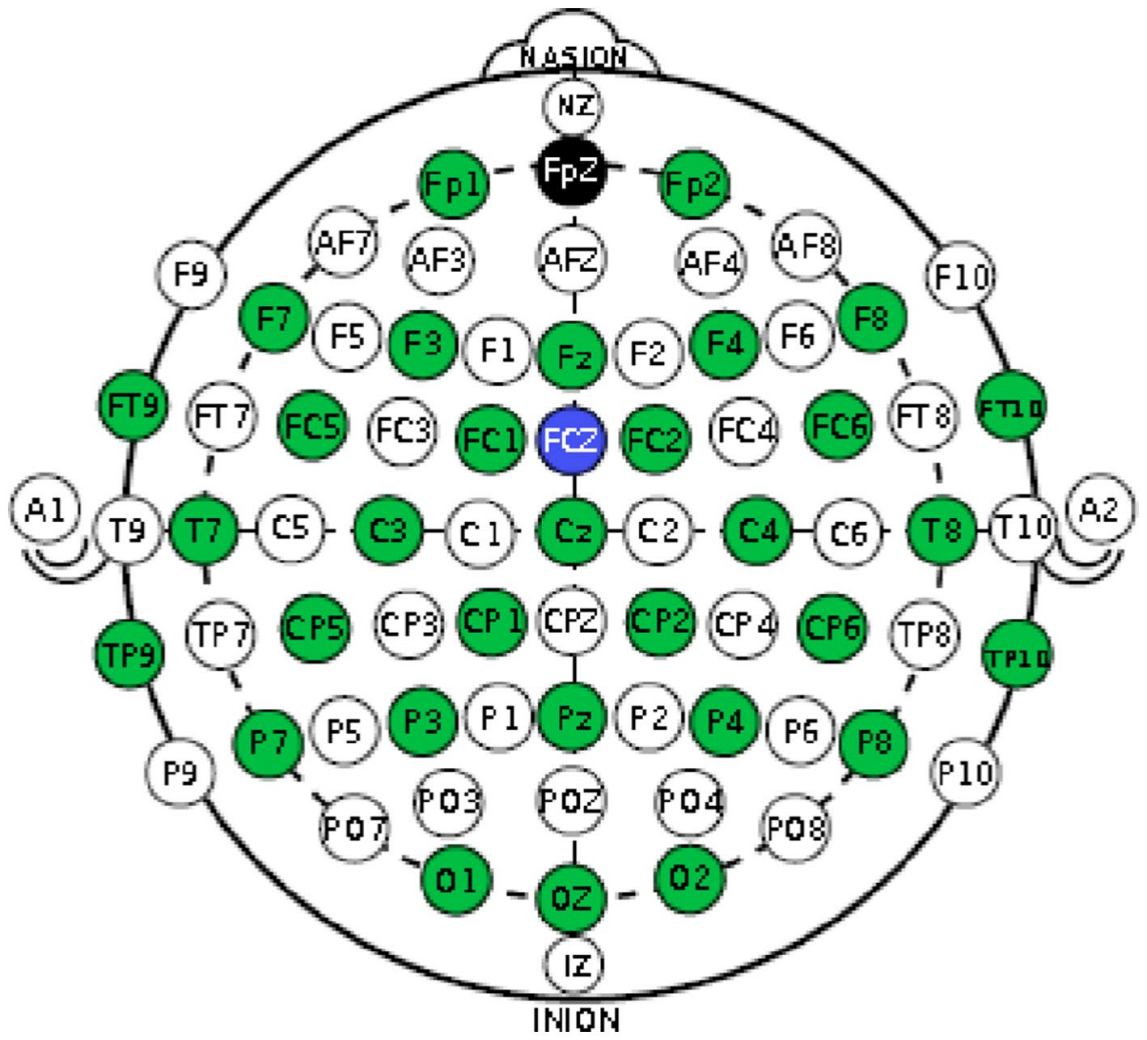
EEG electrode montage showing the positions of the 32 electrodes according to the international 10–20 system. The Fpz electrode served as ground and FCz as reference.

For EEG measurement, a 32-channel active (Ag-AgCl) electrode system was used, following the international 10–20 system. Figure 3 shows the exact positions of these electrodes on the cap. The FpZ electrode served as the ground, while the FCz electrode was used as the reference.

The EEG signals were sampled at a rate of 500 Hz, with the LiveAmp wireless amplifier (Brain Products GmbH, Gilching, Germany). Additionally, the “BrainVision RDA” software redirected the EEG signals to a Lab Streaming Layer (LSL) stream alongside other data streams, such as HR and marker streams. Using the software “LabRecorder” all data streams were synchronously stored into an XDF-formatted file. Preprocessing included bandpass-filtering using a Butterworth filter (order 4), with low and high cutoff frequencies of 1 Hz and 40 Hz, respectively. Common average re-referencing (CAR) step was then performed by subtracting the average across all channels from each channel. A 5-s interval preceding the appearance of the first marker was used for baseline correction, which was applied to the entire signal. Noisy channels were identified using the Pyprep module (Bigdely-Shamlo et al., 2015) by thresholding channels with high deviations. Identified bad channels were removed and interpolated using the MNE library (Gramfort et al., 2014).

HR was measured directly by the VR glasses, which incorporated an integrated optical HR sensor based on a photoplethysmogram (PPG). This sensor illuminated the skin with light emitted by two green LEDs and detected the amount of reflected light, which varies with blood volume changes beneath the skin, thereby enabling HR estimation (Solé Morillo et al., 2022). The resulting PPG waveform reflected the pulse wave of the circulatory system. Using a processing algorithm similar to that described by Siegel et al. (2021), the peak-to-peak intervals of the pulse wave were calculated, enabling the determination of HR. The HR (in bpm) was automatically measured every 5 s and later upsampled to align with the EEG sampling rate.

#### 2.2.3 Questionnaires

The State–Trait-Anxiety Inventory (STAI) questionnaire, developed by Spielberger et al. (1983), was used to assess the general anxiety level of each participant. It distinguishes between state anxiety, which reflects temporary feelings of anxiety at a specific moment, and trait anxiety, representing a person’s habitual tendency to experience anxiety in daily life. The questionnaire consists of 40 items, 20 for each anxiety type, rated on a 4-point Likert scale. The scores for each anxiety dimension range from 20 to 80 points. Participants were categorized into different anxiety groups based on the classification proposed by Kayikcioglu et al. (2017): “no or low anxiety” (20–37 points), “moderate anxiety” (38–44 points) and “high anxiety” (45–80 points). For evaluating arachnophobia the Fear of Spiders Questionnaire (FSQ) was utilized. This questionnaire, based on Szymanski and O’Donohue (1995), consists of 18 items scored on a 7-point scale, with total scores obtained by summing all item points. Although the FSQ lacks a standardized normative score for phobic versus non-phobic populations, Cochrane et al. (2008) divided their participants into three groups based on FSQ scores: low fear (below 33), moderate fear (33–50), and high fear (above 50). This distribution was also used to categorize participants: individuals with FSQ scores above 50 were assigned to high fear (*N* = 7), while those below to 33 were considered as participants with low fear (*N* = 5), the remaining 9 participants were categorized as moderate fear persons. The Brief Mood Introspection Scale (BMIS; Mayer and Gaschke, 1988) was employed to assess current mood. Participants rated 16 adjectives describing mood on a four-point “Meddis” response scale, ranging from “definitely do not” to an unspecified positive end “feel” to “definitely feel.” This scale was useful to see if a specific mood has an influence on the measurement results. This test was then subdivided into four subscores: Pleasant-Unpleasant, Arousal-Calm, Positive-Tired, and Negative-Relaxed Mood. Each of these four subscales contained a different set of adjective items. Details on how these subscales were derived are provided in Mayer and Cavallaro (2019). Furthermore, the overall BMIS mood score was scaled at the end at a scale from −10 to 10.

### 2.3 Experimental procedure

**Figure 4:**
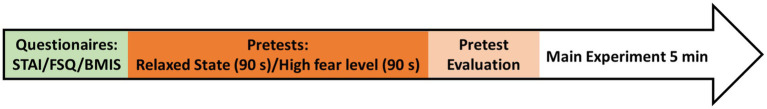
Schematic overview of the experimental procedure. Participants first completed questionnaires assessing anxiety, spider fear, and mood, followed by a calibration (pretest) phase used to train the individualized classifier. Subsequently, participants underwent the main neuroadaptive VR exposure phase, during which EEG and heart rate signals were continuously recorded and used to adapt the spider exposure level in real time based on the estimated fear state.

Initially, participants completed the STAI questionnaire to assess their general anxiety levels (Figure 4). Next, they filled out the FSQ, which specifically measures spider phobia. The final questionnaire was the BMIS. Following the questionnaires, a pretest (i.e., calibration phase) was conducted to record EEG and HR data during both relaxed and spider-stimulated conditions, which were then used to train a classifier to distinguish between the two states.

Next, the HMD was fitted onto the participant’s head and the Unity application was launched. The participant was given time to familiarize themselves with the VR environment and look around. The participant was then positioned so that their gaze was directed toward the edge of the VR setup, where the VR cardboard and shelf would be located—areas where spiders would appear during the experiment. Simultaneously, a Python script was started, which sent out LSL marker streams to mark the exact timing of the experimental paradigm and communicate with the Unity VR environment. Throughout the experiment, participants were asked to minimize movement artifacts by avoiding actions that could introduce noise, such as jaw grinding or excessive blinking.

A prestudy with two participants exhibiting moderate spider fear was conducted to examine potential differences between the left and right frontal hemispheres and to evaluate the feasibility of using FAA index values as features.

In the pretest phase, physiological measurements were collected under two different conditions: a relaxed state and a high spider-fear state. During the relaxed state, the VR environment displayed a neutral scene with no spider stimuli present. Subsequently, the marker stream sent the *Spider_Scene* event, triggering a transition to the fear-inducing scene. After a delay of 5 s, the *Spider_Stimulus* marker was sent to Unity, causing VR spiders to appear across all three stimulus types. Specifically, the environment displayed 100 spiders walking on the floor, 100 spiders walking along the walls, and 5 web-net spiders. All spiders were scaled to the largest main-level size to elicit a maximal fear response. In the pretest phase, participants first spent 90 s in a relaxed scenario, followed by 90 s in the high-fear condition. Following data acquisition, the recorded signals were processed and the data were segmented into epochs and standard signal-processing procedures were applied. For each epoch, the script computed FAA index values for the electrode pairs F3/F4, F7/F8, FC5/FC6, and FT9/FT10, as well as the mean HR. An individualized SVM classifier was then trained to differentiate between no-fear and fear states based on these labeled trials (for more details see section 2.4.1).

During the main experiment the participants were gradually exposed to multiple fear levels based on their current fear state. The fear state of the participant was decoded from physiological data (i.e., EEG and HR) using the individualized SVM classifier. Depending on the output of the classifier the next fear level for the participant was decided. Specifically, if the last fear level was classified as no fear state then the fear stimulus was increased for the participant (i.e., exposure to higher fear level and vise versa). There were five fear levels presented in the VR environment, and the exposure to each level was 30 s. The choice of 30 s duration for exposure was taken from literature (Agres et al., 2023), which supports the fact that an emotional stimulus of 30 s duration can reliably express the desired emotion of the user. Throughout the exposure, participants also provided subjective fear ratings on a scale from 1 (“low fear”) to 5 (“high fear”). These subjective ratings were used offline to label the data and to train and evaluate multiple classifiers in order to assess whether the different exposure levels were discriminable based on physiological signals.

VR stimulus level 1 served as the initial exposure condition. At this level, ten small spiders appeared for both the *walking-on-floor spider* and *walking-along-the-wall spider* stimulus types, while one *web-net spider* was suspended from the ceiling. A loop consisting of 10 iterations was implemented. During each iteration, the program retrieved the most recent 30-s data stream from the EEG and HR measurements. Subsequently, a beep tone was played through the participant’s HMD speakers using the Python module *playsound* (Taylor Marks, 2021). This auditory cue signaled the participant to provide a subjective rating of their current fear level by raising one hand and indicating the rating with a specific number of fingers. One finger represented *no fear*, two fingers *low fear*, three fingers *moderate fear*, and four fingers *high fear*. Following the subjective rating, the Python script executed the signal-processing pipeline, extracted classification features, and predicted whether the VR stimulus level should be increased, decreased, or maintained. Specifically, ratings of *no fear* or *low fear* were intended to trigger an increase in stimulus intensity (number of spiders and size of spiders increased), whereas *moderate fear* or *high fear* ratings were intended to result in a decrease in stimulus intensity. If the classifier output was “*keep stimulus,”* the response was also considered correct when the participant reported *low fear* or *moderate fear*. Summarizing, the fear stimulus intensity is tailored by controlling (1) size of the spider, and (2) number of spiders. Specifically, an environment template with smaller size and less number of spiders is used to convey low level of fear, whereas spider size and number are gradually increased in templates that aim to convey higher levels of fear.

### 2.4 VRSpi

#### 2.4.1 Feature extraction and SVM training

In the pre-test phase, EEG and HR signals recorded during both the relaxed and high-fear conditions were segmented into non-overlapping windows of 500 ms, with each window treated as an individual trial. This resulted in a total of 180 trials per condition and participant (90 s/0.5 s = 180), yielding balanced low- and high-fear datasets for supervised model training. Alpha bandpower values were extracted from EEG using Welch’s method (50% overlap, Hanning window). FAA indices were subsequently computed from the extracted time-varying alpha power values. FAA was derived by subtracting the logarithmic alpha power of the left channel (L) from that of the right channel (R), as expressed by the following formula: FAA = lnR -lnL. FAA indices were calculated from four pairs of symmetric EEG channels, specifically F3/F4, F7/F8, FC5/FC6, and FT9/FT10. These values, acquired from the pretest (i.e., calibration) phase, along with the mean HR within each window were further used to train an SVM classifier.

#### 2.4.2 VRSpi real-time functionality

A total of five VR stimulus levels were used in the experiment, with each higher level introducing more spiders of larger size across all three spider stimulus types. The trained SVM classifier was tasked with determining, based on FAA scores and HR values, whether the VR stimulus level should be increased, decreased, or remain unchanged. Specifically, the classifier provided a binary decision based on the instantaneous physiological indices, determining whether the user would be exposed to the VR environment with increased fear stimulus. In other words, if the user was not fearful, based on his/her physiology then the classifier’s decision led to increased fear stimulus and vice versa. The main experiment was structured as a loop with 10 iterations. In each iteration, the last 30 s of EEG and HR data were pulled from the stream followed by a beep tone signaling that the participant should provide a subjective fear rating by raising a specific number of fingers: One finger for “No Fear”; Two fingers for “Little Fear”; Three fingers for “Moderate/Medium Fear”; Four fingers for “High Fear.” These finger signals were favored over auditory instructions to prevent additional artifacts in the EEG signals caused by vocal responses. The participant had 5 sec to express their rating, which was recorded by the researcher. In a next step the features for classification were extracted and used to predict whether the VR stimulus level should be increased, decreased or maintained. If the participant indicated “No Fear” or “Little Fear,” the classifier should logically increase the stimulus. Conversely, ratings of “Moderate Fear” or “High Fear” should lead to a reduction in stimulus intensity. When the classifier responded with “Keep Stimulus,” it was considered correct if the participant’s rating was “Little Fear” or “Moderate Fear.” The prediction was based on the distance of the current sample features to the SVM model’s hyperplane, indicating whether the features were closer to the relaxed state or the high spider fear state in the pretest feature space (calibration phase). If the distance function for the 30-s window returned an average value below −0.5, indicating the sample belonged to the relaxed state class, the VR stimulus level was increased. Conversely, if the average value was above 0.5, indicating the sample belonged to the fear state class, the stimulus level was decreased. Values between −0.5 and 0.5, within the decision margin, did not trigger any transmission, so the stimulus level remained unchanged for the next iteration. Increasing the VR stimulus level resulted in the introduction of a new spider wave, with the spider size being increased by 10%. On the other hand, decreasing the stimulus level caused the previously introduced VR spiders to disappear.

### 2.5 Offline analysis

To complement the real-time adaptive experiment, we conducted a series of offline, post-hoc analyses aimed at examining neural and physiological responses using participants’ subjective fear ratings as reference labels. These analyses were performed independently of the online adaptive control logic and were intended to characterize fear-related patterns across exposure levels under less restrictive temporal and computational constraints.

#### 2.5.1 FAA analysis

As the subjects confronted the spider scene levels differently based on the classifier predictions during the main experimental paradigm, the average FAA index for each level was calculated using an unequal number of epochs. Only epochs within a specific level were used to calculate its FAA. Statistical analyses were conducted to assess significant differences between the different fear levels using independent t-tests corrected for multiple comparisons. For calculating the FAA index value the average power in the alpha band (8 - 13 Hz) for the channels F3, F4, F7, F8, FC5, FC6, FT9 and FT10 was determined. Then, each opposite channel pair was taken, and the natural logarithm of the left spectrum power was subtracted from the natural logarithm of the right spectrum power to obtain the FAA index value.

#### 2.5.2 HR analysis

In addition to neural features, HR analysis was performed. For each spider exposure scene, HR values were iteratively averaged over 30-s windows, resulting in 10 averaged values per participant. The distribution of these values was compared across the five spider scene levels using independent t-tests. To account for multiple comparisons, *p*-values were adjusted using the Benjamini-Hochberg procedure.

#### 2.5.3 PSD topographical maps

Topographical analyses were conducted assuming that the fear of spiders elicits distinct neural activation patterns, indicative of fear levels,extending beyond reliance on FAA indices. This analysis generated topographical maps to illustrate neural activity across all participants during the spider scene exposure. Bandpowers for all channels were computed and plotted across five frequency bands: Delta (0–4 Hz), Theta (4–8 Hz), Alpha (8–12 Hz), Beta (12–30 Hz), and Gamma (30–45 Hz). Separate topographical maps were created for all frequency bands at the five spider scene levels.

#### 2.5.4 Offline classification

We also conducted an offline, post-hoc classification analysis using EEG data only, as subsequent analyses revealed no significant differences in HR (see section 3.2) across fear levels. This analysis was not intended to drive online adaptation, but rather to assess the separability of different fear levels as subjectively rated by participants and to evaluate the discriminative potential of EEG-derived features under less restrictive computational constraints. Whereas the online adaptation relied on short time windows and low-dimensional, interpretable features to ensure stable and low-latency control, the offline analysis employed longer epochs, different feature representations, and more computationally intensive classifiers to explore which neural features are most informative for fear-state discrimination.

Neural responses during spider scene exposure were captured by segmenting the EEG data into 30-s epochs, each aligned with the duration of a scene. Epochs with excessively high voltages were rejected to mitigate the impact of outliers. The remaining epochs were retained for further analyses and labeled according to the respective spider scene levels. To assess the separability of the different fear levels, we applied frequently used machine learning methods (SVM, RF, and eXtreme Gradient Boosting (XGBoost)) to classify fear levels 1 and 2 against fear levels 4 and 5 as reported by the participants. As level 3 only consisted of 13 trials, assigning these trials to a fear level would not yield reliable results as the dataset would be heavily unbalanced. In total, we extracted 169 30-s long trials. The 2 classes (during high and low spider levels) are selected as “High” including level 4 and 5 (64 trials) and “Low” including level 1 and 2 (105 trials). We extracted the bandpower within the 5 frequency bands and per channel, yielding 160 bandpower values per epoch. Additionally, we derived the FAA indices from F3/F4, F7/F8, FC5/FC6 and FT9/FT10, respectively. In total, there were 164 features created per epoch. Spectral power features were extracted from the physiological signals using the Welch method. The dataset was subsequently normalized using z-score normalization to ensure comparability across features. For model development, the data were split into training and testing sets using an 80%/20% ratio. Hyperparameter optimization was performed via GridSearchCV with three-fold cross-validation on the training data. To assess the robustness and reliability of the classification performance, an outer five-fold cross-validation scheme was employed. Each algorithm was trained independently.

## 3 Results

### 3.1 EEG results

Grand average bandpower topographical maps were computed across five frequency bands (delta, theta, alpha, beta, and gamma) and five fear levels. For the delta band, power was globally high across all levels, with relatively stable patterns and only minor regional differences. In the theta band, widespread cortical activity was observed at all levels, with maximal power over midline and frontal regions. Theta activity showed increases from Level 1 to Level 5, particularly in frontal sites.

For the alpha band, power decreased nonlinearly across levels. At Level 1, alpha activity was distributed broadly over frontal and posterior regions, whereas at higher levels (Level 5) alpha power was reduced, consistent with cortical activation in response to increasing fear intensity. The beta band displayed a similar pattern, with initially moderate power at Level 1, followed by reduced power at Levels 2, especially over central and frontal sites.

**Figure 5:**
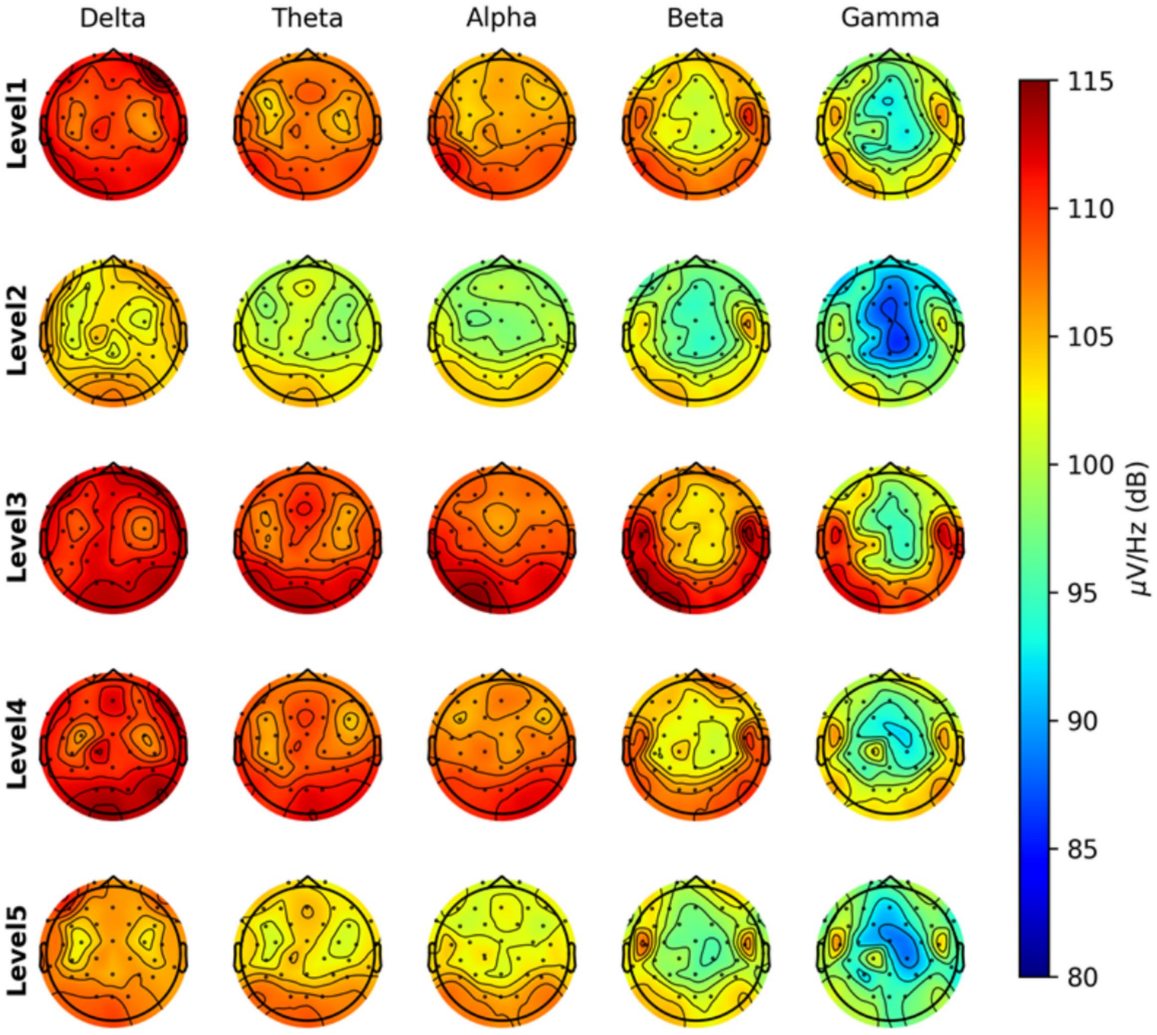
Grand average bandpower topographical maps across five frequency bands (delta, theta, alpha, beta, gamma; columns) and five fear levels (rows). Rows correspond to increasing exposure levels (levels 1–5), while columns represent frequency bands. Color scales indicate relative bandpower magnitude, with warmer colors reflecting higher power. Topographies illustrate spatial distributions of oscillatory activity averaged across participants during spider scene exposure.

In the gamma band, relatively low power was evident at Level 1, with further reductions across Levels 2–5, particularly in central and parietal regions. Gamma suppression was most pronounced at Level 2 and remained attenuated at higher levels (Figure 5).

**Figure 6:**
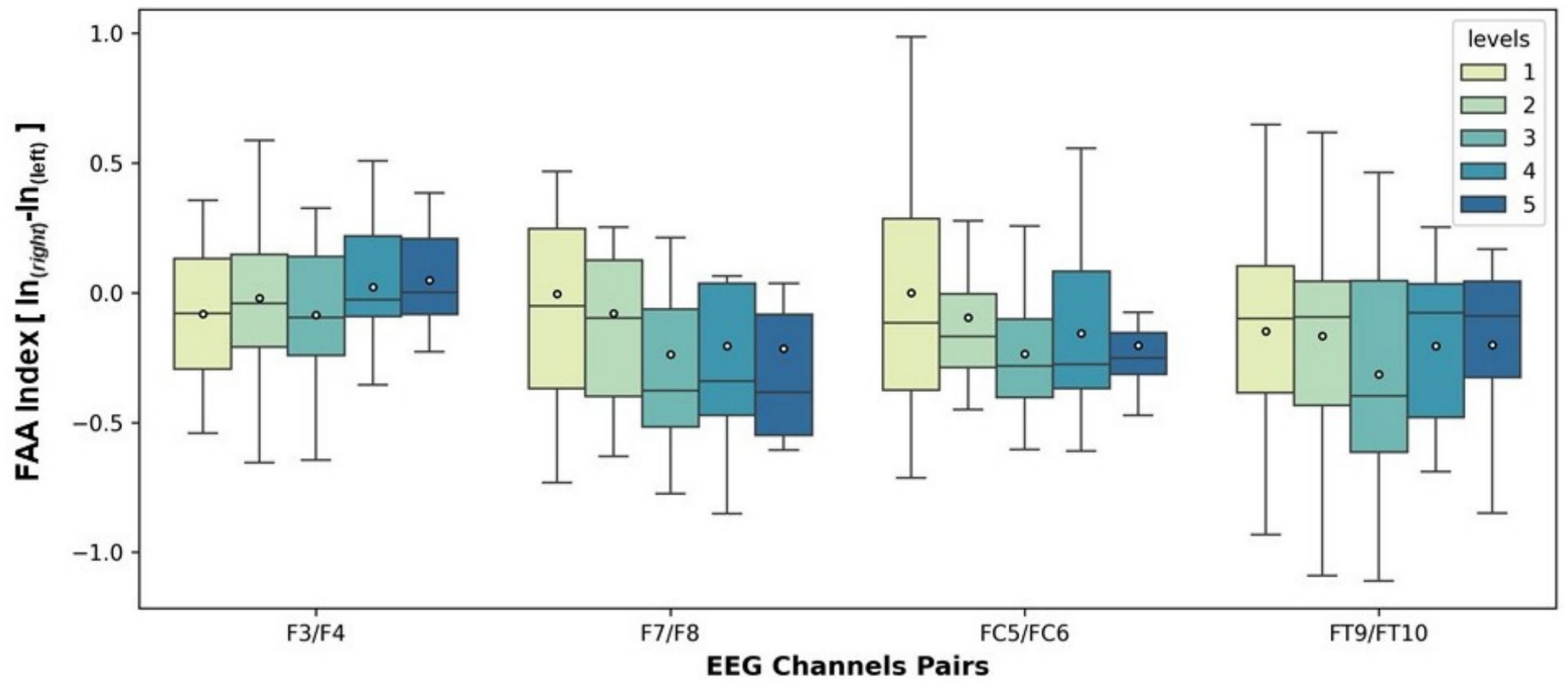
Boxplots of FAA values computed for four symmetric electrode pairs (F3/F4, F7/F8, FC5/FC6, FT9/FT10) across five spider exposure levels. FAA was calculated as the difference between the natural logarithm of right- and left-hemispheric alpha power (FAA = lnR − lnL). The *x*-axis denotes the EEG channel pairs used for FAA computation, while the *y*-axis represents FAA values. Different exposure levels are indicated by distinct boxplots and color coding within each channel pair. Negative FAA values reflect relatively greater right frontal cortical activation. Boxes indicate the interquartile range, the center line denotes the median, and whiskers represent data dispersion.

FAA indices were analyzed across four electrode pairs (F3/F4, F7/F8, FC5/FC6, FT9/FT10) and five spider scene levels. Overall, FAA values tended to be negative across conditions, indicating relatively greater right frontal activity (Figure 6). For the F3/F4 pair, FAA values were slightly negative at most levels, with broader variability at lower scene levels (1–2) compared to higher levels (3–5). For the F7/F8 pair, FAA values were consistently negative across all levels, with a gradual shift toward more pronounced right-sided activity at higher levels (Levels 3–5). For the FC5/FC6 pair, FAA values decreased progressively with increasing scene levels. While Level 1 showed near-neutral values, Levels 3–5 demonstrated more negative FAA indices, indicating a shift toward greater right frontal activation under higher levels of spider fear. Similarly, the FT9/FT10 pair showed a reduction from near-neutral FAA values at Level 1 to more negative values at Level 3, again suggesting a rightward shift in frontal activation as scene intensity increased. Across all channel pairs, variability was greater at lower levels (Levels 1–2), while higher levels showed narrower distributions, indicating more consistent right-lateralized activation.

### 3.2 HR results

**Figure 7:**
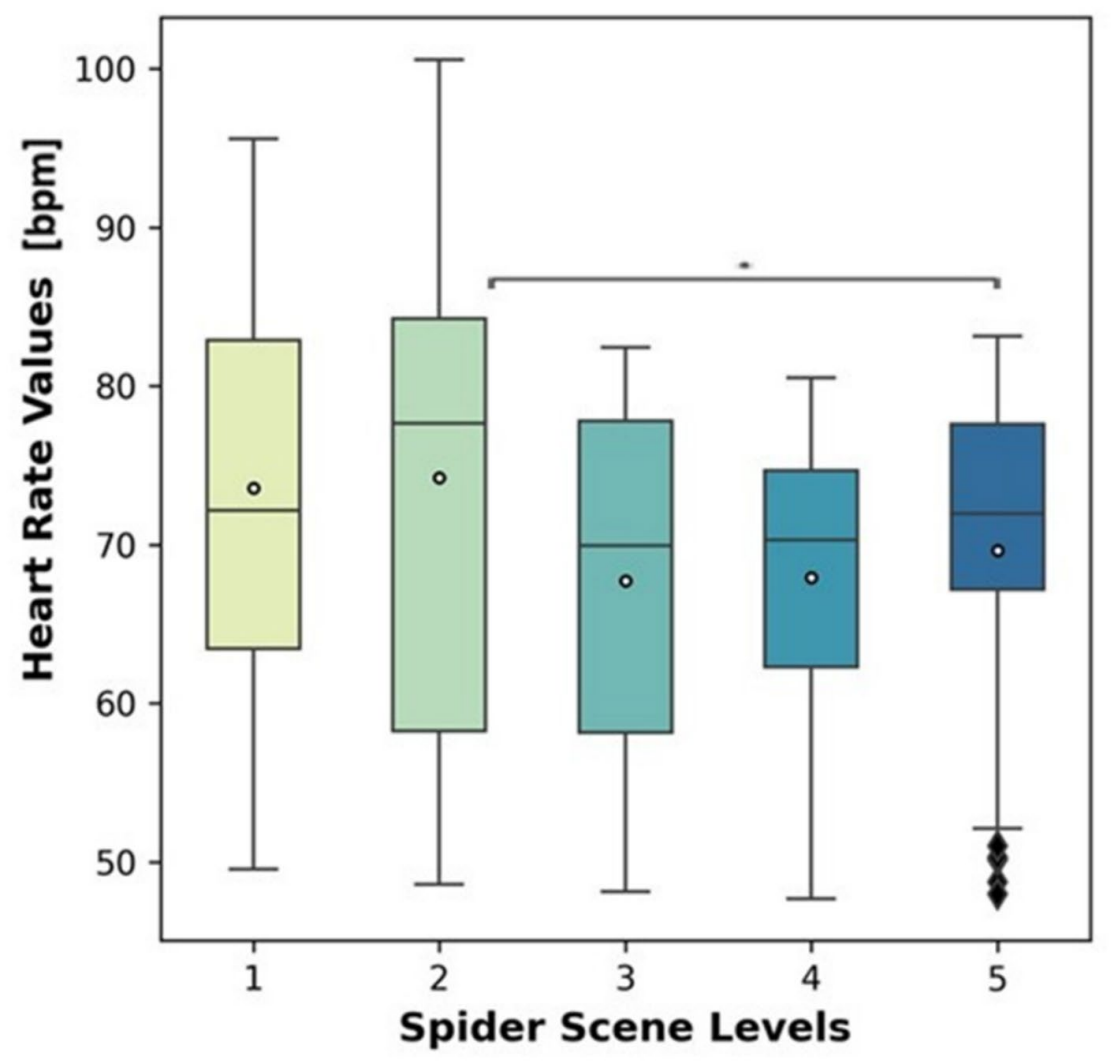
Boxplots of HR (in beats per minute) across five spider exposure levels. Each box represents the distribution of HR values recorded during the corresponding exposure level, with the center line indicating the median, boxes showing the interquartile range, and whiskers denoting data dispersion. Differences in HR across exposure levels illustrate non-linear cardiovascular responses during increasing exposure.

Figure 7 presents HR distributions across five spider scene levels, representing increasing levels of fear exposure. Median HR appears slightly elevated at Levels 1 and 2 (around the mid-70s), with somewhat lower medians at Levels 3 and 4 (~70 bpm), and a modest rise again at Level 5 (~72 bpm). Several low outliers appear at Level 5, reflecting individuals with unusually low HR responses in this condition. Independent t-tests with Benjamini-Hochberg correction revealed no significant pairwise differences between exposure levels. Specifically, HR decreased from the initial exposure (Level 1) to the most intense scene (Level 5), contrary to what might be expected if fear responses simply increased linearly with scene intensity.

### 3.3 Correlations between neurophysiology and subjective measures

The following section presents a series of correlation matrices examining potential associations among EEG features, HR, and questionnaire measures. All correlations were tested two-tailed and corrected for multiple comparisons using the Benjamini–Hochberg procedure (*alpha* < 0.05).

**Figure 8:**
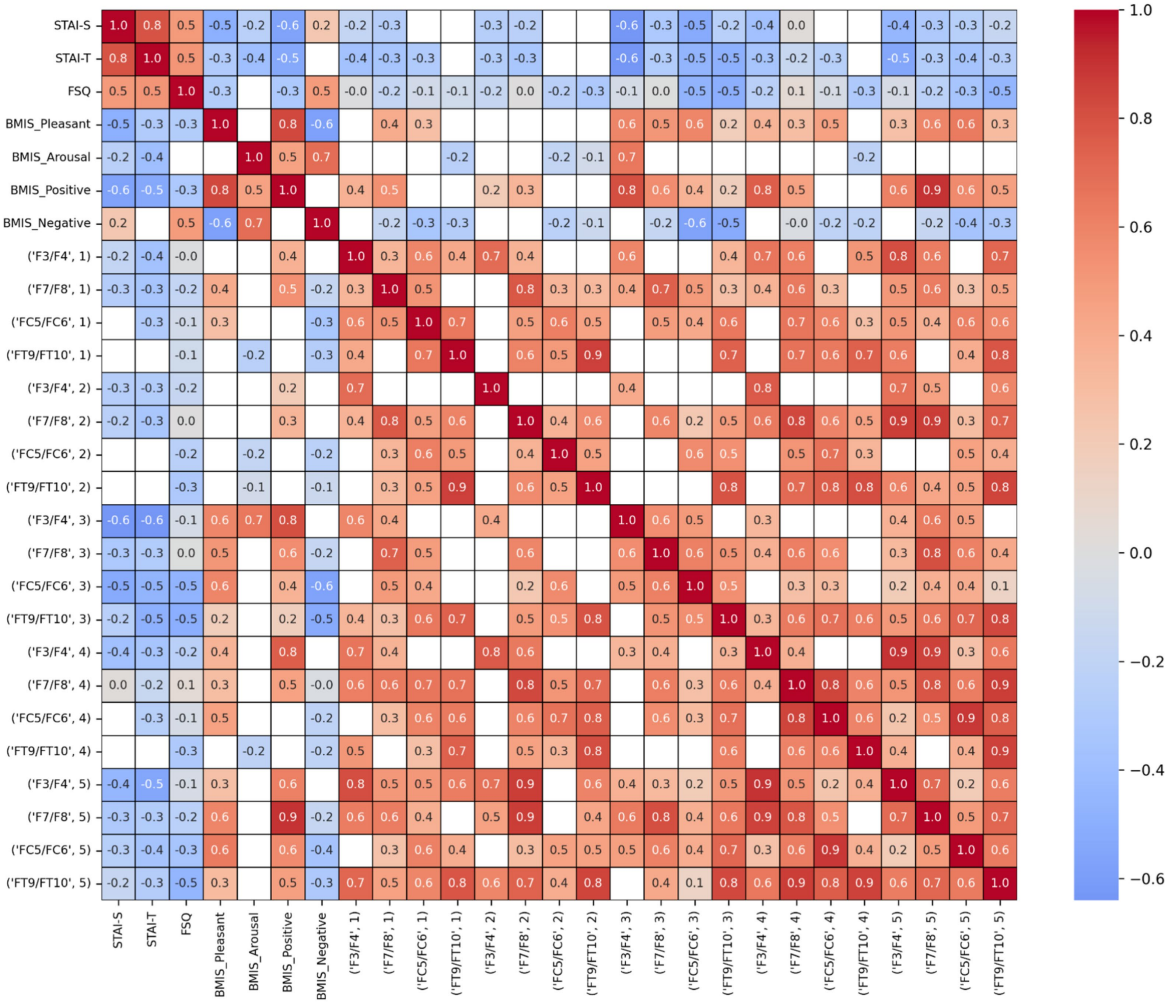
Correlation matrix showing relationships between self-report questionnaire scores (state and trait anxiety, spider fear, and mood subscales) and EEG-derived features, including FAA indices. Color intensity indicates the strength and direction of correlations (blue: positive, red: negative). This matrix illustrates associations between psychological measures and neurophysiological indices across participants.

To illustrate the strength and direction of monotonic relationships between pairs of variables, we calculated a Pearson correlation matrix (Figure 8) with Benjamin-Hochberg correction for multiple comparisons. In the figure only significant relationships are coloured, while non-significant pairs are masked in white. It shows how strongly and in which direction the ranked values of one variable (e.g., EEG features) are related to the ranked values of another variable (e.g., questionnaires), across all pairs of variables in the dataset. The correlation matrix revealed several notable patterns across self-report questionnaires and EEG-derived measures.

State and trait anxiety were strongly positively correlated (STAI-S–STAI-T: *r* = 0.80), indicating substantial overlap between situational and dispositional anxiety. The FSQ was moderately positively correlated with both STAI-S (*r* = 0.50) and STAI-T (*r* = 0.50), suggesting that FSQ captures anxiety-related variance while remaining partially distinct from the STAI scales. The BMIS subscales showed a clear and theoretically coherent affective structure. BMIS Pleasant was strongly positively correlated with BMIS Positive (*r* = 0.80) and strongly negatively correlated with BMIS Negative (*r* = −0.60). BMIS Positive and BMIS Negative were also strongly negatively associated (*r* = −0.60). BMIS Arousal correlated positively with both BMIS Positive (*r* ≈ 0.50) and BMIS Negative (*r* ≈ 0.70), consistent with arousal reflecting emotional activation rather than affective valence. Anxiety measures were systematically associated with mood. STAI-S, STAI-T, and FSQ showed moderate negative correlations with BMIS Pleasant and BMIS Positive (r range = −0.30 to −0.60), and moderate positive correlations with BMIS Negative (*r* range = 0.40 to 0.50), indicating that higher anxiety was associated with lower positive affect and higher negative affect. EEG features exhibited moderate to strong positive correlations for different frontal and fronto-temporal electrode pairs (F3/F4, F7/F8, FC5/FC6, FT9/FT10), indicating substantial spatial coherence of the EEG measure across frontal regions. Frontal EEG features were moderately positively associated with positive mood states. Across electrodes and fear levels, correlations with BMIS Pleasant and BMIS Positive generally ranged from *r* ≈ 0.40 to 0.60, with stronger associations observed in higher levels. In contrast, EEG features showed weak-to-moderate negative correlations with anxiety measures, including STAI-S, STAI-T, and FSQ (*r* range = −0.30 to −0.60). These findings suggest that higher anxiety was associated with reduced expression of the frontal EEG feature.

**Figure 9:**
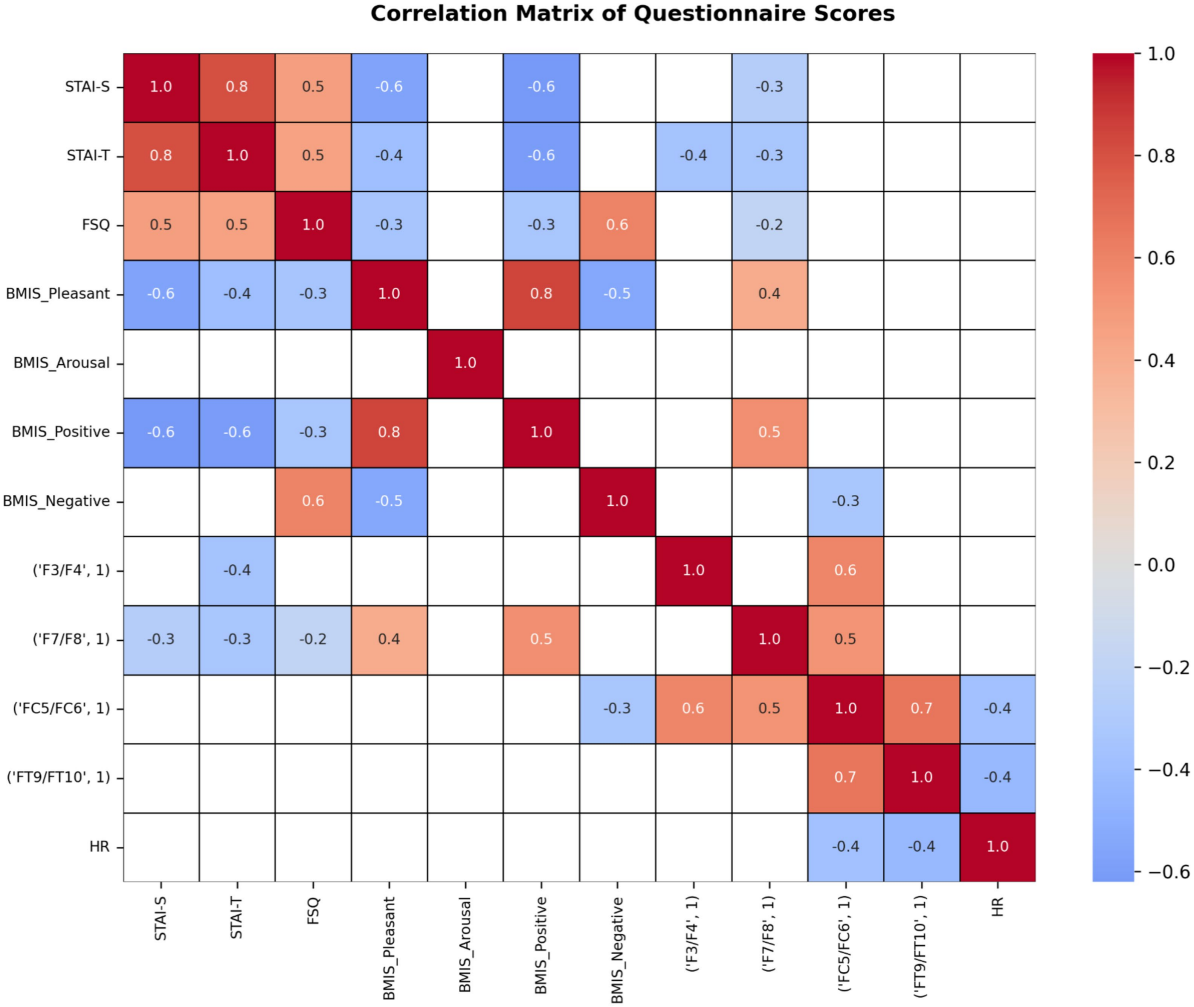
Correlation matrix illustrating relationships between questionnaire measures (state and trait anxiety, spider fear, and mood subscales), EEG-derived features (FAA), and HR at the lowest spider exposure level (fear level 1). Color intensity represents the strength and direction of correlations (blue: positive, red: negative), highlighting associations among psychological and physiological variables under low fear levels.

**Figure 10:**
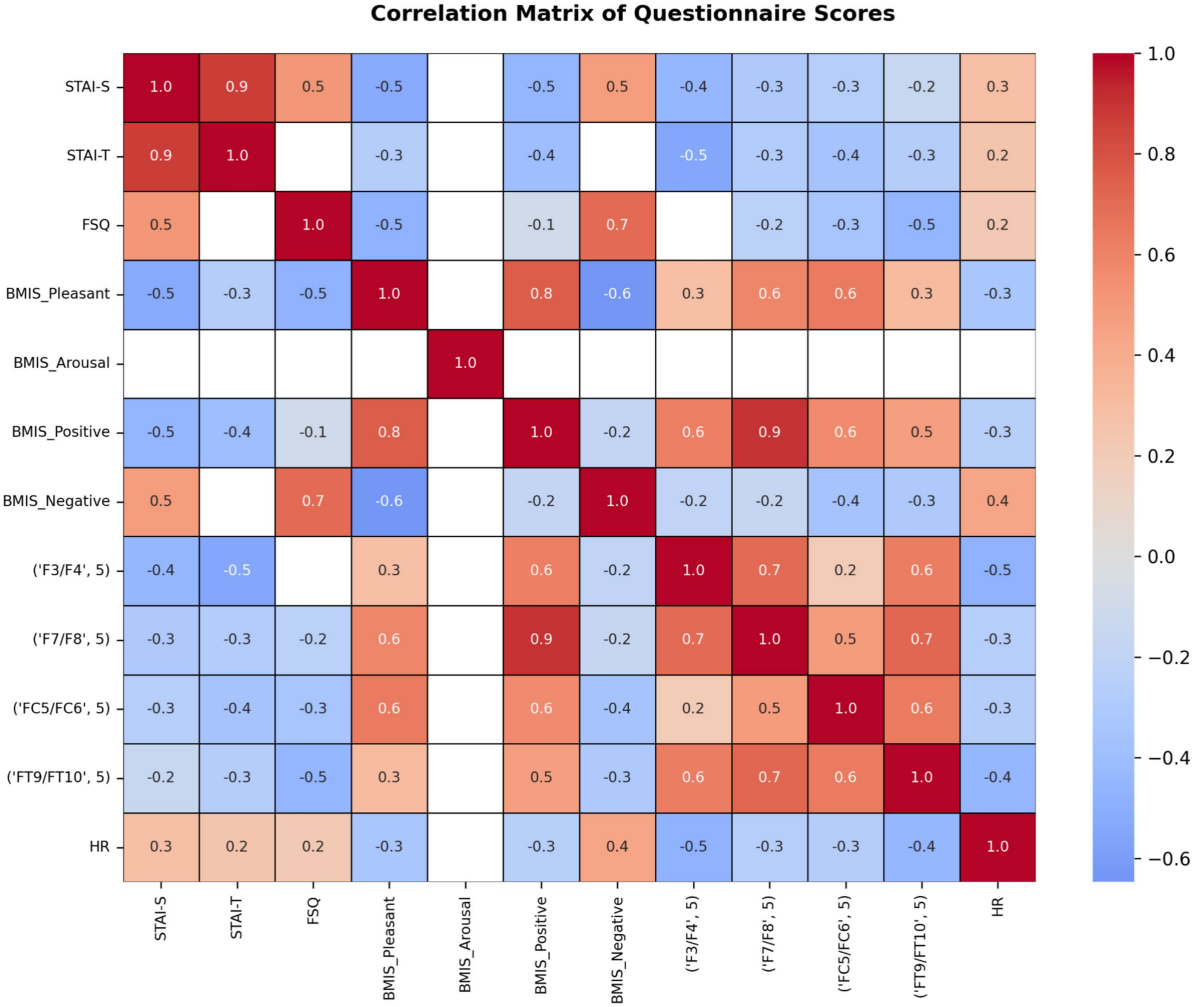
Correlation matrix illustrating relationships between questionnaire measures (state and trait anxiety, spider fear, and mood subscales), EEG-derived features (FAA), and HR at the highest spider exposure level (fear level 5). Color intensity represents the strength and direction of correlations (blue: positive, red: negative), highlighting associations among psychological and physiological variables under high fear levels.

To examine the relationships between physiological measures such as HR, neural correlates, and subjective scales in greater detail, Pearson correlation matrices were computed separately for the lowest fear level (Level 1; Figure 9) and the highest fear level (Level 5; Figure 10). A Benjamin-Hochberg correction was applied for correction of multiple comparisons. Only significant correlations are indicated.

For fear level 1 (Figure 9), state and trait anxiety were strongly positively correlated (STAI-S–STAI-T: *r* = 0.80), indicating substantial overlap between situational and dispositional anxiety. The FSQ showed moderate positive correlations with both STAI-S and STAI-T (*r* = 0.50 for both), suggesting partial convergence with established anxiety measures. Mood subscales from the BMIS demonstrated a coherent affective structure. BMIS Pleasant was strongly positively correlated with BMIS Positive (*r* = 0.80) and moderately negatively correlated with BMIS Negative (*r* = −0.50). BMIS Positive was also strongly negatively associated with anxiety measures (STAI-S and STAI-T: *r* = −0.60 for both). BMIS Negative showed a moderate positive correlation with FSQ (*r* = 0.60) and a negative association with BMIS Pleasant (*r* = −0.50). These patterns indicate that higher anxiety is associated with reduced positive affect and increased negative affect. Frontal EEG asymmetry indices showed moderate associations with mood measures. Asymmetry at F7/F8 was moderately positively correlated with BMIS Pleasant (*r* = 0.40) and BMIS Positive (*r* = 0.50), suggesting greater relative frontal activity was associated with more positive affect. This measure also showed weak negative correlations with anxiety measures (STAI-S and STAI-T: *r* = −0.30). Asymmetry at FC5/FC6 was positively correlated with asymmetry at F3/F4 (*r* = 0.60) and F7/F8 (*r* = 0.50), indicating spatial coherence across frontal regions. FC5/FC6 asymmetry was negatively correlated with BMIS Negative (r = −0.30), suggesting an inverse relationship with negative affect. Heart rate was moderately negatively correlated with frontal EEG asymmetry at FC5/FC6 and FT9/FT10 (*r* = −0.40 for both), indicating that higher heart rate was associated with reduced frontal EEG asymmetry. No substantial correlations were observed between HR and self-report measures. Overall frontal EEG asymmetry indices showed moderate agreement across sites and were modestly associated with affective valence, with greater frontal asymmetry related to more positive and less negative mood. Associations involving EEG and heart rate were small to moderate.

For fear level 5, correlation analyses (Figure 10) revealed strong associations between anxiety, mood, and physiological measures. State and trait anxiety were very strongly positively correlated (STAI-S–STAI-T: *r* = 0.90), indicating substantial overlap between situational and dispositional anxiety. FSQ was moderately positively correlated with STAI-S (*r* = 0.50) and strongly positively correlated with BMIS Negative (*r* = 0.70), suggesting that higher fear-related symptoms were associated with increased negative affect. BMIS Pleasant was strongly positively correlated with BMIS Positive (r = 0.80) and strongly negatively correlated with BMIS Negative (*r* = −0.60). BMIS Positive was moderately to strongly negatively associated with anxiety measures (STAI-S: *r* = −0.50; STAI-T: *r* = −0.40) and showed a small negative association with FSQ (*r* = −0.10). In contrast, BMIS Negative was moderately positively correlated with STAI-S (*r* = 0.50) and HR (*r* = 0.40), indicating that greater negative affect was associated with higher anxiety and increased physiological arousal. Frontal EEG asymmetry indices exhibited strong positive intercorrelations across electrode pairs, including F3/F4 with F7/F8 (*r* = 0.70) and FT9/FT10 (*r* = 0.60), as well as F7/F8 with FT9/FT10 (*r* = 0.70). EEG asymmetry measures were moderately associated with mood states. Asymmetry at F7/F8 showed strong positive correlations with BMIS Positive (*r* = 0.90) and BMIS Pleasant (*r* = 0.60), while asymmetry at F3/F4 was moderately positively correlated with BMIS Positive (*r* = 0.60). Across electrode sites, EEG asymmetry measures were weakly to moderately negatively correlated with anxiety measures (STAI-S and STAI-T: r range = −0.20 to −0.50), suggesting that higher anxiety was associated with reduced frontal EEG asymmetry. HR showed moderate negative correlations with frontal EEG asymmetry indices (F3/F4: *r* = −0.50; FT9/FT10: *r* = −0.40), indicating that higher HR was associated with lower frontal EEG asymmetry. HR was weakly positively correlated with anxiety measures (STAI-S: *r* = 0.30; STAI-T: *r* = 0.20) and negatively correlated with BMIS Pleasant and BMIS Positive (*r* = −0.30 for both).

Overall, the results demonstrate strong and theoretically consistent relationships among anxiety and mood self-report measures, robust correlations among frontal EEG asymmetry indices, and modest associations between frontal EEG activity, affective valence, and physiological arousal. Greater frontal EEG asymmetry was associated with more positive and less negative affect, whereas higher anxiety and heart rate were linked to reduced frontal EEG asymmetry. Given the modest sample size these findings should be interpreted cautiously and considered as a proof of concept study.

#### 3.3.1 Comparison of fear level 1 and 5 correlation patterns

If we take a closer look at the lowest and highest fear levels and its neurophysiological and psychological associations some diverse results are observed. Pearson correlation analyses were conducted separately for Level 1 and Level 5 to examine relationships among self-reported anxiety and mood, frontal EEG asymmetry, and HR. Across both levels, questionnaire measures demonstrated highly consistent and theoretically coherent patterns, whereas associations involving EEG and physiological measures varied as a function of fear level.

At both Level 1 and Level 5, state and trait anxiety were very strongly positively correlated (Level 1: *r* ≈ 0.80; Level 5: *r* = 0.90), indicating stable convergence between situational and dispositional anxiety. The FSQ showed moderate positive correlations with anxiety and negative affect (*r* range ≈ 0.50–0.70) and negative correlations with positive and pleasant affect (r range ≈ − 0.30 to −0.50) at both levels. Similarly, BMIS Pleasant and BMIS Positive were strongly positively correlated (*r* ≈ 0.80), and both were strongly negatively correlated with BMIS Negative (*r* ≈ −0.50 to −0.60). These findings indicate that the affective and anxiety structure of the self-report measures was robust and invariant across levels, supporting the reliability and construct validity of the questionnaire data.

In contrast to the stability of self-report measures, associations involving frontal EEG asymmetry differed in magnitude across levels. At Level 1, EEG asymmetry indices showed weak-to-moderate intercorrelations and modest associations with mood and anxiety measures (typically *r* ≈ 0.30–0.50). At Level 5, however, EEG asymmetry indices exhibited stronger spatial coherence across frontal sites (e.g., inter-electrode correlations up to *r* ≈ 0.70), suggesting increased consistency of frontal neural activity. Importantly, EEG–affective associations were substantially stronger at Level 5. Frontal asymmetry, particularly at F7/F8, showed strong positive correlations with positive mood (*r* = 0.90) and pleasant affect (*r* = 0.60), whereas corresponding relationships at Level 1 were weaker. Across electrode sites, EEG asymmetry was also more strongly negatively correlated with anxiety at Level 5 (up to *r* = −0.50) than at Level 1. These results suggest that frontal EEG asymmetry more clearly reflects affective valence and anxiety under higher-level conditions, potentially indicating increased emotional engagement or enhanced sensitivity of neural markers at Level 5.

HR showed minimal or inconsistent associations with self-report and EEG measures at Level 1. In contrast, at Level 5, HR demonstrated moderate negative correlations with frontal EEG asymmetry (*r* ≈ −0.40 to −0.50), as well as positive correlations with negative affect (*r* = 0.40) and negative correlations with positive and pleasant affect (*r* ≈ −0.30). This pattern suggests stronger coupling between physiological arousal, affective experience, and frontal neural activity at the higher level.

Taken together, the results indicate that while psychological constructs of anxiety and mood remain stable across levels, the relationships between neural, physiological, and affective measures are level-dependent. Specifically, higher-level conditions are associated with stronger coupling between frontal EEG asymmetry, affective valence, and physiological arousal. This pattern is consistent with models proposing that brain–behavior relationships become more pronounced under conditions of increased emotional or cognitive engagement.

Given the modest sample size, these findings should be interpreted cautiously. Nonetheless, the convergence of stronger EEG–mood, EEG–anxiety, and EEG–HR associations at Level 5 suggests that frontal EEG asymmetry may serve as a more sensitive marker of affective state under higher-demand or higher-intensity conditions.

### 3.4 Offline classification results

As a complement to the real-time adaptive experiment, we conducted an offline, post-hoc classification analysis to assess the separability of fear levels and the discriminative potential of EEG-derived features. The offline classification results are shown in Table 1 including classification accuracies and the corresponding F1 scores for the binary discrimination between low fear (Levels 1 and 2) and high fear (Levels 4 and 5). In general, all classification accuracies ranged from 67.45 to 87.65%. The overall best performance was given with the SVM algorithm using only PSD values (i.e., bandpower features) or combining PSD with FAA. Using only FAA index values as features achieved a maximum of 75.29%. However, using PSD with and without FAA index values resulted in accuracies ranging from 74.99 to 87.65%. Throughout the different algorithms, a remarkable difference in classification performance was observed in XGBoost, with the lowest performance observed when using PSD and FAA separately.

**Table 1:** Offline classification performance for distinguishing between low fear (levels 1 and 2) and high fear (levels 4 and 5) spider exposure conditions using different feature sets and machine learning algorithms.

	PSD		FAA		PSD + FAA	
	Acc	F1-score	Acc	F1-score	Acc	F1-score
SVM	87.65 ± 5.70	87.47 ± 5.65	75.29 ± 5.76	74.99 ± 5.27	87.65 ± 6.28	87.55 ± 6.18
Random Forest	78.82 ± 6.55	78.77 ± 6.59	71.18 ± 5.70	69.64 ± 5.83	77.06 ± 5.06	76.93 ± 5.06
XGBoost	79.41 ± 5.58	79.48 ± 5.50	68.24 ± 6.55	67.45 ± 6.43	81.18 ± 8.44	81.10 ± 8.46

Reported metrics include classification accuracy and F1 score averaged across cross-validation folds. Results are shown for models trained on PSD-based features (i.e., bandpower), FAA features, and their combination, highlighting the contribution of different neurophysiological feature sets to fear-state discrimination.

The feature importance revealed the top features as theta power from Pz, beta and gamma activity from Fp2 as well as beta and gamma power from C4 with RF. For the XGBoost the most important features were gamma power at TP10 and CP1, theta from Pz and beta from P3, T8 and CP1. Considering using only FAA values, the most important electrode pairs were F7/F8 and FC5/FC6 for both algorithms. In the combined approach (Bandpower + FAA), F7/F8 was following the previously mentioned band power features as 7th most important feature of the RF, whereas no FAA value occurred in the 10 most important features for XGBoost.

## 4 Discussion

The present study first introduced a novel VRET for spider fear (VRSpi) including neurophysiological features of the user and second analysed related psychological and neurophysiological responses while acting in the neuroadaptive virtual environment VRSPi. By combining self-report measures, HR indices, and FAA, we aimed to characterize how fear responses evolve with increasing exposure intensity.

### 4.1 Subjective measures

At both fear levels, state and trait anxiety were strongly correlated, but this relationship strengthened under higher fear intensity, consistent with the view that situational distress amplifies trait-like vulnerability (Endler and Kocovski, 2001). At lower levels, anxiety was primarily expressed as a reduction in positive affect, whereas at higher levels it was more directly associated with negative affect. This shift supports theories of affective experience in which anxiety progresses from absence of positive mood toward active negative affect under escalating threat (Watson and Clark, 1994). FSQ scores also showed stronger links to negative mood at higher levels, indicating that stress/fear indices became more predictive of subjective emotional experience with increased fear levels.

### 4.2 FAA results

The findings indicate that increasing confrontation with spider scenes elicited a rightward shift in FAA, particularly in lateral (F7/F8) and centrofrontal (FC5/FC6, FT9/FT10) regions. This pattern is consistent with theories linking greater right frontal activation to withdrawal-related affect, avoidance motivation, and anxiety (Davidson, 1992; Coan and Allen, 2004; Thibodeau et al., 2006). The reduction in variability at higher scene levels suggests that participants exhibited a more uniform neurophysiological response when exposed to the most intense spider scenes, reflecting convergence toward a defensive withdrawal state. It should be noted though that these effects were observed in a non-clinical sample, and the magnitude and temporal dynamics of FAA shifts may differ in individuals with clinically diagnosed arachnophobia.

### 4.3 Correlation results

The present findings indicate that the relationship between psychological and physiological measures varies as a function of fear intensity. At Level 1, Anxiety at Level 1 was characterized by reduced positive affect and modest associations with negative affect, consistent with models that emphasize the inverse association between anxiety and positive mood states (Watson and Clark, 1994). In contrast, at Level 5, state and trait anxiety were more strongly coupled and more strongly associated with negative mood, suggesting that heightened task demands amplify the overlap between trait vulnerability and situational distress (Endler and Kocovski, 2001). FSQ scores became more predictive of negative affect at Level 5, with a novel positive correlation with pleasant mood, suggesting that fear-related arousal may elicit mixed affective experiences depending on context (Barrett, 2006). Physiological indices also showed condition-dependent changes. EEG intercorrelations were stronger at Level 5, reflecting increased neural synchrony under cognitive load (Harmony, 2013). HR showed moderate negative associations with frontal EEG asymmetry at Level 5. It also displayed modest associations with mood only at Level 5, in line with evidence that autonomic indices become more tightly integrated with subjective affect during heightened stress (Thayer et al., 2012). Taken together, these findings suggest that higher fear intensity promotes greater integration across psychological and physiological systems, with anxiety shifting from an absence of positive affect toward a more direct expression of negative mood and arousal.

### 4.4 HR results

The observed pattern of HR responses across spider scene levels suggests that cardiovascular activity is not linearly related to fear intensity. HR was initially elevated at lower scene levels, which may reflect novelty effects and anticipatory anxiety when first confronted with fear-relevant stimuli (Bradley et al., 2001; Grillon, 2002). As exposure intensified, HR decreased, consistent with habituation processes whereby repeated presentations of aversive stimuli lead to reduced physiological reactivity (Thompson and Spencer, 1966; Domjan, 2005). At the highest level (Level 5), HR was significantly lower than at Level 2, a finding that aligns with the defensive “freezing” response, characterized by parasympathetic-mediated bradycardia during sustained or intense threat (Fanselow, 1994; Hagenaars et al., 2014; Roelofs, 2017). Together, these findings support the view that fear responses are dynamic, shifting from anticipatory arousal to adaptive inhibition as threat exposure escalates.

Summarizing cardiovascular data revealed a nonlinear trajectory. HR was elevated during initial exposures, likely reflecting novelty and anticipatory anxiety (Bradley et al., 2001; Grillon, 2002). With repeated or intensified exposure, HR decreased significantly, particularly from Level 2 to Level 5. This pattern suggests habituation of autonomic responses (Thompson and Spencer, 1966; Domjan, 2005) and aligns with research linking intense threat to parasympathetic dominance and defensive freezing responses (Fanselow, 1994; Hagenaars et al., 2014; Roelofs, 2017). Thus, the cardiovascular system appears to shift from early arousal to later defensive inhibition under sustained fear.

Neurophysiological indices corroborated this defensive profile. Across multiple frontal and frontocentral channel pairs, FAA values became increasingly negative as confrontation intensity rose, indicating greater right frontal activation. This pattern is consistent with models associating right-lateralized activity with withdrawal-related affect, avoidance motivation, and anxious states (Davidson, 1992; Coan and Allen, 2004; Thibodeau et al., 2006). Notably, variability in FAA decreased at higher fear levels, which may suggest a convergent neural response across participants during the most intense scenes.

The bandpower results indicate changes in cortical dynamics that may reflect greater cortical activation and reduced synchronization with increasing fear levels (Figure 5). The reduction in alpha power across levels could be associated with heightened attentional engagement and arousal (Klimesch, 1999), while decreases in beta and gamma power may reflect increased cognitive load and emotional processing demands (Başar et al., 2001; Güntekin and Başar, 2010). In contrast, theta power progressively increased, especially in frontal regions, possibly reflecting heightened vigilance, emotional reactivity, and regulatory effort. The modest increase in frontal theta power furthermore may suggest enhanced working memory and emotional regulation processes under higher fear (Cavanagh and Frank, 2014). Delta activity remained relatively stable across levels. Taken together, these findings indicate that escalating spider scene exposure is associated with reduced oscillatory power in faster bands (alpha, beta, gamma) and increased frontal theta activity, potentially indicating a neural state characterized by heightened vigilance, emotional reactivity, and regulatory effort.

These findings highlight that fear responses are dynamic and integrative. Psychological data show a shift from reduced positive affect to active negative mood, while physiological systems reveal complementary patterns: initial cardiovascular arousal gives way to defensive bradycardia, and frontal EEG asymmetry demonstrates consistent right-lateralized activation. This convergence across modalities suggests that with increasing exposure, fear states become more coherent, uniform, and dominated by withdrawal-related processes. However, as participants were not clinically phobic, cardiovascular responses in a clinical population may be more pronounced or follow different temporal patterns.

### 4.5 Offline classification

In addition, we performed a separate offline analysis to probe how well different fear levels could be distinguished based on both neural activity during real-time adaptive control. An overall good performance further supports the distinct separability of the fear levels. All classifier types yielded similar results, but varied in performance depending on the used features. Using only FAA index values yielded an accuracy of 68.24%, whereas performance increased when using only PSD features (87.65%). Combining the two feature sets did not lead to notable changes in accuracy, especially in SVM. As discussed in the previous section, oscillatory power changes occurred not only in the alpha band but also in beta and gamma bands, as well as in frontal theta activity. This indicates that the FAA index from frontal electrode pairs, as a standalone feature, does not capture the full range of information about the emotional state. In contrast, bandpower features covering the whole scalp appear to contain more relevant information. This interpretation is supported by the feature importance analysis, which highlighted central/parietal electrodes in the theta band and frontal/central electrodes in the beta and gamma bands as most informative. When compared with recent studies (Arsalan and Majid, 2021; Aldayel and Al-Nafjan, 2024), our results align with current findings and further demonstrate XGBoost as a robust method for fear classification. Moreover, we showed that using FAA index values alone as an indicator of stress levels performs less effectively than including PSD features, particularly from parietal and central electrode sites.

### 4.6 Limitations and future directions

This study has several limitations that should be addressed in future work. First, the sample size was small, which limits the statistical power of the analyses and precludes drawing firm conclusions regarding significant effects and constrains the strength of inferences that can be drawn from EEG and HR measures. Nevertheless, the main emphasis of this work was on developing the VR environment and study design rather than on formal hypothesis testing.

Second, the gender distribution was imbalanced, with a predominance of male participants. This is particularly relevant because women are more likely to develop spider phobia than men (Schmitt and Müri, 2009). Future studies should therefore aim to recruit a larger proportion of female participants to better reflect the population most affected by arachnophobia.

Third, none of the participants met criteria for a clinical arachnophobia diagnosis, but instead reported only moderate fear of spiders. This likely reduced the strength of neurophysiological differentiation between neutral and spider-related scenarios. In clinical populations, stronger and more distinct neural markers of fear (e.g., FAA shifts) would be expected. Although the VR environment allowed for controlled and repeatable fear induction, participant reports indicated that the perceived intensity of the stimuli was not uniformly sufficient across individuals, highlighting limitations in ecological validity and suggesting that subjective presence and realism may modulate both emotional and physiological responses.

A future follow-up study should test the system in individuals with clinically significant arachnophobia, ideally across multiple sessions, and evaluate treatment outcomes using behavioral measures such as the Behavioral Approach Test (BAT) administered pre- and post-therapy.

Fourth, while FAA results suggested the possible influence of emotional regulation strategies (e.g., cognitive reappraisal), this was not directly assessed. Including questionnaires such as the Emotion Regulation Questionnaire (ERQ; Gross and John, 2003) would help clarify whether individual differences in reappraisal use are indeed associated with positive FAA shifts.

A further limitation of the present study is the absence of a non-neuroadaptive or sham-adaptive control condition. Without such a comparison, it is not possible to conclusively attribute the observed behavioral and physiological changes solely to the adaptive component of the system. Future studies should include appropriate control conditions to disentangle the specific contribution of neuroadaptive exposure from general VR exposure effects.

In addition, the current real-time adaptation relied on a binary fear-state formulation (low vs. high fear). While this representation enabled reliable and low-latency adaptive control in this proof-of-concept implementation, fear responses are inherently dynamic and continuous. This binary formulation may therefore oversimplify the temporal evolution of emotional states. Future extensions of the framework may incorporate multi-level or continuous fear representations to better capture fear dynamics while preserving robustness and real-time performance.

Another limitation concerns the distinction between the features used for online adaptive control and those evaluated in offline classification analyses. The apparent discrepancy between the FAA-based features employed for real-time adaptation and the higher classification performance achieved by PSD features in offline analyses reflects an intentional design choice. FAA was selected initially for online use due to its strong theoretical grounding in affective neuroscience, low dimensionality, interpretability, and computational efficiency, which are critical for stable and low-latency real-time adaptation. In contrast, offline classification analyses were designed to explore the discriminative potential of richer feature sets under less restrictive computational and timing constraints. Accordingly, superior offline classification performance does not necessarily translate to optimal real-time adaptive behavior. Future work will therefore systematically compare feature sets in online settings.

Another important aspect is that several participants reported that the VR environment did not feel sufficiently realistic to induce fear. VR offers a high degree of experimental control and safety, it may not fully replicate the sensory richness or perceived threat of real-world spider encounters for all participants. Improving ecological validity could involve transitioning from VR to augmented reality (AR), which offers greater presence by combining virtual spiders with the user’s real environment (Juan et al., 2005). In addition, multimodal sensory stimulation could be introduced. For example, Brice et al. (2021) used an ultrasonic haptic feedback system to simulate the sensation of a spider crawling on the hand, while Kurscheidt et al. (2019) employed vibrotactile arm sleeves to mimic spiders moving along the arms. Such approaches could enhance immersion and evoke more authentic fear responses.

An additional limitation concerns the characterization of the real-time adaptive feedback loop itself. Although the system operates online and modulates exposure based on ongoing EEG and heart rate signals, the present study does not systematically evaluate key control-related properties such as detection latency, temporal stability, or potential feedback oscillations. At this stage, the adaptive framework is intended as a proof-of-concept that enables investigation of how neurophysiological markers evolve during adaptive exposure, rather than as a fully validated real-time control system. Future work should therefore include dedicated analyses of adaptive timing, responsiveness to rapid physiological changes, and the impact of feedback delays on user experience and physiological regulation. Such evaluations will be essential for understanding the dynamic behavior of neuroadaptive exposure systems and for ensuring their reliability in more clinically oriented, multi-session applications.

Beyond technical development, future work should also consider requirements for clinical integration of neuroadaptive exposure systems. In clinical settings, EEG-based adaptations would need to demonstrate sufficient reliability, stability across sessions, and robustness to artifacts to be trusted by therapists as a decision-support tool rather than a replacement for clinical judgment. Establishing conservative adaptation thresholds, transparent decision logic, and clinician override options may be essential for real-world deployment. While the present work focuses on feasibility, addressing these practical considerations will be critical for translating neuroadaptive VRET frameworks into usable therapeutic tools.

## 5 Conclusion

We presented the design of a VR-based neuroadaptive, individualized VRET system (VRSpi) for treatment of arachnophobia with a special focus on the underlying neurophysiological and psychological correlates of the users. VRSpi is designed to present varying fear stimuli (spider stimulus) real-time based on the instantaneous physiological manifestation (EEG and HR) of the participant. The real-time SVM classifiers are able to determine the next exposure level for the participants, and thus can be used to provide a graded and progressive exposure therapy to arachnophobic individuals. In addition, detailed analyses of brain activity, HR, and anxiety-related questionnaires provide deeper insights into the underlying neurophysiological and psychological responses. Taken together, our findings highlight that fear responses are dynamic and integrative. Psychological data show a shift from reduced positive affect to active negative mood, while physiological systems reveal complementary patterns: initial cardiovascular arousal gives way to defensive bradycardia, and frontal EEG asymmetry demonstrates consistent right-lateralized activation. This convergence across modalities suggests that with increasing exposure, fear states become more coherent, uniform, and dominated by withdrawal-related processes which might improve existing VRET systems relying solely on subjective reports (e.g., questionnaires, self-ratings). Therefore VRSpi delivers an innovative solution by incorporating real-time EEG and HR indices, enabling objective monitoring of fear states with a more accurate and dynamic picture of a patient’s response.

## Data Availability

The raw data supporting the conclusions of this article will be made available by the authors, without undue reservation.
