# Supplementary material for: VRSPi: towards a neuroadaptive VR exposure therapy system for spider phobia
Source: Front Hum Neurosci. 2026 Mar 4;20:1717588. doi: 10.3389/fnhum.2026.1717588 (PMC12996115; doi:10.3389/fnhum.2026.1717588)
Supplement: Supplementary file 1 [file Table_1.DOCX]

|  | PSD | | FAA | | PSD + FAA | |
| --- | --- | --- | --- | --- | --- | --- |
|  | Acc | F1-Score | Acc | F1-Score | Acc | F1-Score |
| SVM | 87.65 ± 5.70 | 87.47 ± 5.65 | 75.29 ± 5.76 | 74.99 ± 5.27 | 87.65 ± 6.28 | 87.55 ± 6.18 |
| Random  Forest | 78.82 ± 6.55 | 78.77 ± 6.59 | 71.18 ± 5.70 | 69.64 ± 5.83 | 77.06 ± 5.06 | 76.93 ± 5.06 |
| XGBoost | 79.41 ± 5.58 | 79.48 ± 5.50 | 68.24 ± 6.55 | 67.45 ± 6.43 | 81.18 ± 8.44 | 81.10 ± 8.46 |
